# A narrative review of proactive palliative care models for people with COPD

**DOI:** 10.1177/17534666241310987

**Published:** 2025-02-08

**Authors:** Amy Pascoe, Xinye Chen, Natasha Smallwood

**Affiliations:** Respiratory Research @ Alfred, School of Translational Medicine, Monash University, Melbourne, VIC, Australia; Respiratory Research @ Alfred, School of Translational Medicine, Monash University, Melbourne, VIC, Australia; Department of General Medicine, The Alfred Hospital, Melbourne, VIC, Australia; Respiratory Research @ Alfred, School of Translational Medicine, Monash University, East Block level 2, 55 Commercial Rd, Melbourne, VIC 3004, Australia; Respiratory and Sleep Medicine, The Alfred Hospital, Melbourne, VIC, Australia

**Keywords:** chronic obstructive pulmonary disease, models of care, palliative care

## Abstract

Chronic obstructive pulmonary disease (COPD) refers to a group of lung diseases that are distinct in underlying aetiology but share a common disease course of persistent and progressive airflow restriction. People living with COPD, as well as the people who care for them, frequently have severe and unmet physical and psychosocial needs, including breathlessness, fatigue, cough, anxiety and depression. Early proactive palliative care is well placed to address these needs, yet it is frequently under-utilised in this group. This narrative review aimed to identify core components of palliative care and examine how existing models of care are implemented to better understand which models can best serve the needs of people with COPD. Symptom palliation, advance care planning, and support for caregivers emerged as the common components underpinning both generalist and specialist models of palliative care. Models of proactive palliative care were diverse in terms of where and how care was delivered as well as which health professionals were involved. Five key models of palliative care were identified: (1) multi-disciplinary integrated services, (2) nurse-led care, (3) hospice and residential aged care, (4) home-based care, and (5) telemonitoring and telehealth. Each model describes a diverse set of interventions and many of these share common elements, including the normalisation of palliative principles within routine care and the provision of diverse delivery settings to accommodate individual preferences and needs. Successful palliative care models must be practical, accessible and innovative to respond to individuals’ complex and evolving needs, foster multi-disciplinary collaboration and input and optimally utilise local healthcare resources.

## Introduction

### Prevalence, symptom burden, and unmet patient and carer needs in advanced chronic obstructive pulmonary disease

Chronic obstructive pulmonary disease (COPD) refers to a group of lung diseases that are distinct in underlying aetiology but share a common disease course of persistent and progressive airflow restriction.^1^ The global prevalence of COPD was estimated at 392 million cases in 2019 with three quarters of these occurring in low- or middle-income countries.^2^ These numbers are projected to grow to 600 million by 2050 and to disproportionately impact women and people living in low- or middle-income countries.^3^

People living with advanced COPD frequently experience severe and distressing symptom burdens, including breathlessness (also called dyspnoea), fatigue, cough, anxiety and depression, social isolation, and overall reduced quality of life.^1^ The burden of COPD can also impact the physical and psychological health of people who provide formal or informal care for people with advanced COPD, typically friends or family members.^4,5^ Optimal disease-directed management of the underlying condition is often insufficient to adequately address the physical, emotional, and psychosocial needs of people with advanced COPD or the people who care for them.^6^ Consequently, even amongst those who are able to access timely and high-quality disease-directed care, many will experience severe and prolonged unmet symptom needs.^7,8^

Palliative care is well placed to address the unmet needs of people living with advanced COPD, as well as the needs of the people who care for them. Despite this, international audits have demonstrated that fewer than 20% of decedents with COPD receive palliative care referrals.^9–11^ and these are often late in the illness trajectory when the ability for shared decision-making and planning is limited.^12^

### Professional consensus: current state of international guidelines and recommendations

The benefits of palliative care implemented early in the illness trajectory and integrated with existing generalist or specialist respiratory services are increasingly recognised in international clinical practice guidelines for the management of COPD.^13–17^ The Global Strategy for the Diagnosis, Management, and Prevention of COPD (GOLD report)^16^ is a regularly updated comprehensive document developed by an international collaboration of clinicians and public health experts that informs all aspects of COPD management. The 2024 GOLD report highlights inequity in palliative care access for people with COPD and details specific palliative approaches which should be considered not only at the immediate end of life but also to support symptom management throughout the illness trajectory.^16^

International professional respiratory societies have similarly championed the need for palliative care with their own position statements. The American Thoracic Society (ATS) in partnership with a number of multi-disciplinary societies with interests in palliative care developed a 2022 position statement regarding integration of palliative care early in the care continuum for people with serious respiratory illness and provides a framework to support this goal.^14^ The framework developed by ATS and partner organisations spans models of care, holistic symptom management, formalised discussion and documentation of goals of care, consideration of health inequities and carer needs, and responsiveness to crises.^14^ The European Respiratory Society (ERS) has also called for consideration of palliative care that is integrated into routine respiratory care to support the physical, psychological, social and existential needs of people with COPD as well as their carers in a 2023 clinical practice guideline.^13^ This guideline specifically acknowledged that although the evidence base consistently favoured conditional recommendations for palliative care, it was underpinned by studies that were of low to very low certainty, reflecting the lack of robust studies on the topic.^13^ A further clinical practice guideline published in 2024 by the ERS addresses strategies for symptom-directed care for people with non-malignant chronic respiratory disease, including COPD, and recommends referral to multi-component, multi-disciplinary, symptom management services.^15^ This recommendation highlights the potential of holistic and integrated care to improve patient outcomes with low associated healthcare expenditure and low risk to patients.^15^

These recommendations from leading professional societies are also reflected in many government policy documents which name palliative care as a national priority in the management of chronic conditions. In Australia, a 2019 report outlining the national strategic framework for chronic conditions, including COPD, makes mention of palliative care under both the need for active engagement, which focuses on empowering patients to be shared decision makers in their care, as well as the need for accessible health services, which includes alternative models of delivery outside of traditional hospital services.^18^ In the United Kingdom and United States, the National Institute for Health and Care Excellence (NICE) and the Department of Defence Veterans Affairs have each published guidelines specific to COPD that recommend multi-disciplinary care, including palliative care specialists.^19,20^

### Generalist or specialist palliative care approach

Palliative care is needed throughout the disease trajectory of COPD and should be accessible early and concurrently with disease-directed therapies.^21,22^ Palliative care services are now available in both hospital-based and community-based settings, including inpatient consultation or palliative care beds, outpatient specialist clinics, residential and aged care facilities, general practices and patients’ homes.^21,23^ The Australian Palliative Care Service Development Guidelines stated that all health professionals who care for people with life-limiting diseases should be trained to have basic competencies in palliative care, including symptom management and discussions about prognosis and goals of treatment.^21,24^ This was also strongly recommended in the ATS statement on palliative care for patients with respiratory diseases in 2008.^22^ When palliative care is provided by general practitioners, nurses, allied health professionals or medical specialists in other disciplines, it is referred to as ‘generalist palliative care’, ‘primary palliative care’ or ‘palliative care’.^21,25,26^

In contrast to ‘generalist’ palliative care, ‘specialist palliative care’ is comprised of a multi-disciplinary and multi-professional team of palliative care physicians, nurses and allied health professionals who have advanced skills, extensive experiences and training in providing palliative care to address more complex needs, issues or conflicts in a wide range of hospital-based and community-based settings.^26,27^ In addition, they also have roles in providing education, support and consultation to facilitate ‘generalist palliative care’.^28,29^

With the growing population living with chronic life-limiting illnesses and increasing needs for palliative care, a collaborative approach between specialist and generalist palliative care teams was proposed by *Quil and Abernethy* to expand the reach of palliative care services and empower clinicians from any medical discipline to proactively practice basic palliative care skills and integrate them into routine practices.^24^ There are not enough palliative care specialists to meet the increasing demand for palliative care. Therefore, other medical specialists need to practice basic palliative care skills to make palliative care more accessible for patients and facilitate delivery of generalist palliative care.^24^ This collaborative mixed model would encourage bidirectional education opportunities, enhance the interdisciplinary partnership and improve access to palliative care and healthcare costs.^30,31^

### Barriers to accessing palliative care

Despite guidelines recommending early palliative care approaches as routine practice for patients with advanced COPD, access to palliative care services is limited, delayed and fragmented.^32^ Several barriers have been identified related to patients, health professionals and systems. The unpredictable disease trajectory and lack of reliable prognostic tools make it challenging to prepare for sudden deterioration and introduce palliative care promptly.^33,34^ Patients and families receive little education about the disease’s nature and symptom palliation and are often not prepared for end-of-life care.^34^ More importantly, this is a particularly vulnerable patient cohort who often experience multiple comorbidities, severe symptom burden, low socioeconomic status, psychosocial isolation and diverse geographical locations, which may prevent them from accessing timely and regular palliative care.^32^

With multiple healthcare professionals (general practitioners, specialist physicians, nurses, allied health professionals, etc.) involved in COPD management, there is a lack of clarity in terms of who is responsible for initiating palliative care discussions and coordinating various services and appointments.^35^ Unfortunately, without effective communication and coordination amongst health professionals, important aspects of patient-centred care and needs are neglected, compromising efficiency and continuity of care. Time constraints within clinical consultations and lack of training in palliative care for healthcare professionals are also limiting factors that reduce the quality of palliative care delivery and lead to poor therapeutic relationships.^32,34,36,37^

Furthermore, people with COPD have unique palliative care needs in physical, psychological and social domains, which are different from people with malignancy or other chronic conditions, and therefore may not respond to standard palliative care management.^13^ Currently, there is no unified definition of the palliative care approach for COPD, nor are there consistent referral pathways that effectively integrate palliative care into the standard disease-directed management. The provision of palliative care is limited by the funding, resources and expertise available in the local healthcare systems. Therefore, the establishment of proactive integrated palliative care models needs to be innovative and practical to optimise the utilisation of local resources, strengthen team ownership, respond to diverse patients’ and carers’ needs and be readily accessible through flexible locations and modes of care delivery.

## Aims and rationale

The evidence documenting palliative care needs amongst this group is well-established. What remains to be understood is how best to deliver palliative care to extend the reach of existing limited specialist resources, improve the understanding and acceptability of palliative care as a mode of supportive care that is not restricted to immediate end-of-life, and ultimately improve access to high-quality symptom-supportive care throughout the illness trajectory for people with COPD and the people who care for them. This review aims to examine core components of palliative care and existing models of proactive and integrated palliative care which leverage both specialist and generalist skillsets to better understand which models can best serve the needs of people with COPD.

## Methods

This narrative review sought an overview of the relevant literature by searching MEDLINE for English-language original research reports, narrative reviews, and systematic reviews related to palliative care for people with COPD and multi-disciplinary models of care that integrated palliative care components. We only reviewed models of care that delivered palliative care management. In addition, we sought relevant clinical practice guidelines from international professional societies to describe current expert consensus on the topic. Thematic headings were developed from a broad reading of the literature with key reports described under each heading. References from reports of interest were also considered.

## Proactive palliative care approaches

**Figure 1.**
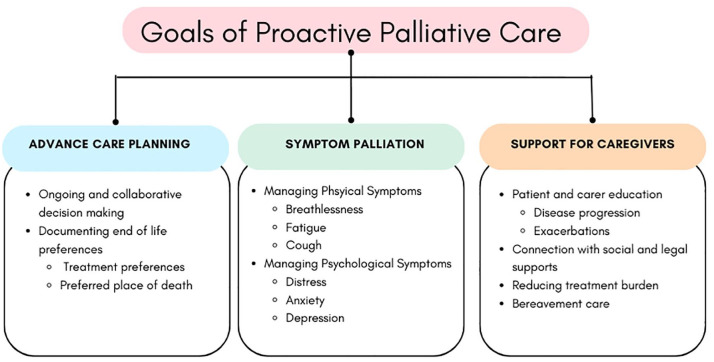
Goals of proactive palliative care for people with COPD. COPD, chronic obstructive pulmonary disease.

This section will describe the common goals of proactive palliative care that are frequently described in models of care for people with COPD (Figure 1).

### Advance care planning

Advance care planning (ACP) is an integral part of palliative care and should be introduced early into routine practice. Discussion and documentation of advanced care planning encourages people to share their ‘personal values, life goals, and preferences regarding future medical care’ with health professionals and trusted ones and ensures that the medical care they receive aligns with their wishes.^38^ Important topics for consideration during ACP include the progressive nature of COPD, treatment preferences during hospital admissions (e.g. specifically wishes regarding receiving non-invasive ventilation, invasive ventilation or resuscitation), patient’s and carers’ understanding and preferences, and what end-of-life care might look like (particularly place of preferred death).^39^ Importantly ACP is an iterative process, not only because it is difficult to cover everything in one discussion, but people’s preferences and views often evolve over time and may completely reverse.^40^ Thus offering repeated opportunities to discuss ACP is critical. Effective ACP can avoid unnecessary, aggressive, life-sustaining treatment and hospitalisation, allow early palliative care planning and improve quality of end-of-life care with higher patient satisfaction and less surprises.^41,42^ More importantly, ACP eases anxiety, guilt and conflict amongst caregivers to make rushed decisions at the time of sudden deterioration.^39,43,44^

However, the initiation of ACP conversations can be challenging and are often delayed and poorly communicated. Several barriers have been identified, including unpredictable prognostication, time constraints to discuss ACP during a busy clinic consultation, lack of care coordination and continuity, limited training of healthcare professionals in ACP discussion and/or communication skills, and cultural and spiritual factors.^36,40,45^ Importantly, patients often report having a limited understanding of COPD and express a desire for open and honest conversations with clinicians in order to make individualised and informed decisions.^37^ Unfortunately, many ACP discussions are initiated during acute exacerbations in hospitals with unfamiliar doctors in a chaotic environment.^46^ This may add unnecessary distress to patients and carers and affect timely communication between secondary and primary care.^47^ Moreover, health professionals find it challenging to engage end of life care (EOLC) conversations due to lack of training and clarity regarding their roles and responsibilities in ACP initiation and follow-up.^36,48^

To optimise the quality of ACP discussion and respond to patients’ needs, there is an increasing role for generalist palliative care in the community, particularly for patients in regional and rural settings.^31,49^ Patients with advanced COPD may benefit from community or home-based care programmes that can overcome psychosocial or geographical barriers and encourage their ongoing engagement.^50^ A collaborative approach between general practitioners with specialist physicians, nurses and allied health professionals can facilitate an accessible and sustainable palliative care model.^31^

### Symptom palliation

Patients with advanced COPD often suffer from multiple comorbidities, leading to severe symptom burden, psychological distress and functional impairment.^8^ Breathlessness is the most prominent and debilitating symptom experienced by patients.^51,52^ It is a subjective feeling that varies individually and may predict poor prognosis.^53,54^ However, it is often under-recognised and under-treated due to insidious symptom onset and poor understanding of disease trajectory.^55^ Therefore, patients tend to normalise and adapt to the symptom by restricting their daily activities.^52^ Chronic breathlessness syndrome is defined as severe and persistent breathlessness despite optimised disease-directed therapy,^56^ which requires a multi-disciplinary approach to integrate both non-pharmacological and pharmacological interventions.^17,57^ Non-pharmacological interventions should be prioritised and optimised first with early referral to pulmonary rehabilitation, education on breathlessness exercise, and written self-management plans.^58^ In addition, singing has also been trialled in patients with advanced respiratory conditions and has shown positive effects on well-being, enjoyment, social engagement and self-management.^59,60^ These strategies are greatly valued by patients and caregivers.

In regard to pharmacological management, low-dose (<30 mg daily) sustained-release oral opioids may be safe and effective to relieve refractory breathlessness.^61,62^ However, it is worth noting that current guideline recommendations are based on trials of stable outpatients, but opioids are frequently initiated in the inpatient setting after an acute exacerbation with lack of opioid education and delayed follow-up.^63^ This is a particularly vulnerable group to treat. Therefore, careful initiation, slow titration, thorough education and prompt outpatient review are highly recommended to ensure safe opioid use in the community.^63^

Furthermore, cough and fatigue are highly prevalent among patients with COPD. They commonly co-exist with breathlessness and complicate its management.^64^ The burden of chronic cough is under-estimated and often normalised to be the consequence of smoking and disease progression.^65^ Many factors, including physical and psychosocial, interact with fatigue, making it more challenging to provide effective symptom relief. The underlying aetiology and clinical assessments of fatigue remain unclear and heterogeneous.^66^ To optimise symptom relief with tailored treatment, detailed screening of symptoms and their impact on quality of life and families should be integrated into routine practice.^66^ Further research in comprehensive assessment tools and evaluation of their complex interaction is needed to gain a better understanding of symptom burden and to develop targeted treatment.^64^

### Support for caregivers

Caring for patients with COPD can be demanding and cause burnout, but carers have received little attention, resulting in significant physical, occupational and psychosocial burdens and negative family dynamics.^67^ Carers play an essential role in improving treatment adherence, reducing exacerbations and supporting symptom palliation at home.^68^ However, with increasing care dependency and responsibilities, carers have reported experiences of anxiety, helplessness, social isolation, worries for future uncertainty, and fear to discuss end-of-life treatment decisions.^69^ As COPD patients may die suddenly and unexpectedly, bereavement support should be accessible and actively provided to all carers and families to reduce their anxiety, guilt and post-traumatic grief.^70^

## Existing palliative care models for advanced COPD

**Figure 2.**
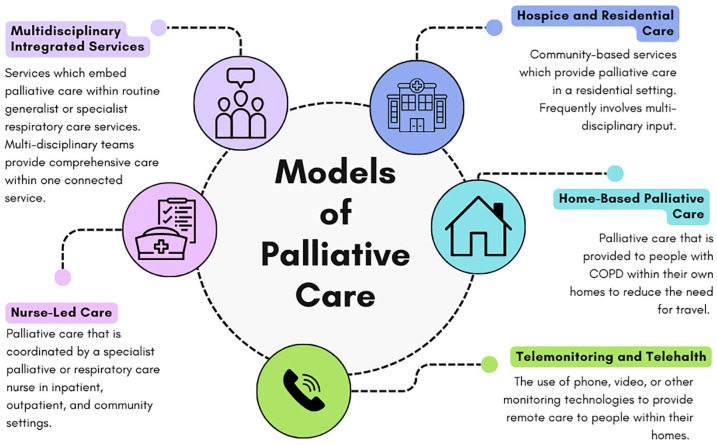
Models of palliative care for people with COPD. COPD, chronic obstructive pulmonary disease.

This section will describe the key models of care that have been implemented and evaluated for people with COPD (Figure 2). These models describe distinct but often overlapping components that aim to expand access to palliative care.

### Multi-disciplinary integrated services

There has been increasing emphasis on integrating palliative care into standard disease-directed optimisation approaches for people with non-malignant respiratory diseases. Multiple models of multi-disciplinary, multi-professional, single point-of-access services have emerged since the late 20th century^71–75^ (Table 1). These services consist of palliative care and/or respiratory specialists for the initial face-to-face disease and symptom assessment, followed by regular counselling and review by nurses and allied health professionals via home visits or telephone consultations. The evidence-based non-pharmacological interventions have become an essential part of education and self-management, such as pulmonary rehabilitation, handheld fans, airway clearance and pacing techniques, cognitive behaviour therapy and psychosocial support.^17^ These interventions may be offered together by an integrated breathlessness service to target the complex interaction between symptoms (breathlessness, anxiety, depression, fatigue, etc.) and improve symptom palliation.^71–75^ Written information and crisis action plans tailored to individuals’ needs are also provided to patients and carers.^76^ The team meets regularly to discuss individualised treatment plans and work closely with other medical specialists, primary care practitioners and community services (hospital support teams, rehabilitation programmes, aged care assessment, dietitians, community palliative care and hospices) to identify any gaps and ensure sustainable patient-centred care.^71–75^

**Table 1.** Summary of multi-disciplinary integrated services.

Service	Service duration	Country	Conditions	Settings	Healthcare professionals	Interventions	Study duration	Quantitative outcomes	Qualitative outcomes	Cost-effectiveness
Cambridge Breathlessness Intervention Service^73,79,80^	2003–ongoing	UK	Chronic breathlessness regardless of the underlying causes	Predominantly in patients’ homes, or outpatient visits in person/ by phone	Palliative care consultants, OT, PT and an administrator	Breathlessness and symptom assessment; non-pharmacologial and pharmacological management; breathing exercises and aids; reassurance and education of self-management; individualised written action plans; psychological support; nutrition; referrals to other specialists, psychologists, rehabilitation and hospice services; liaison with GPs and community services	RCT for 4 weeks	Non-statistical improvements in patients and carers’ distress due to breathlessness; CRQ mastery scores in chronic breathlessness, patients and carers’ anxiety and depression scores	Reduced fear, anxiety and fear; improved confidence in breathlessness management; appreciation of the service	Slightly higher inpatient costs (£100) after excluding the outliers
Breathlessness Support Service^71,81,82^	2010–2012	UK	Chronic breathlessness with mMRC ⩾2 regardless of the underlying causes	Predominantly outpatient clinic weekly, combined with home visits and phone calls	Joint palliative care and respiratory specialists, clinical nurse specialists, OT, PT and SW	Optimisation of disease-directed management; non-pharmacological and pharmacological breathlessness management; education of self-management and crisis plans; home assessments and provision of aids; psychosocial assessment	RCT for 6 weeks	Significant improvement in the CRQ mastery of breathlessness; non-statistical improvements in activities of daily living, breathlessness on exertion and depression	Improved confidence, function, and control over breathlessness	No significant difference
INSPIRED^74,83,84^	2010–ongoing	Canada	COPD	Predominantly home based, combined with outpatient and phone calls	Respiratory therapist educators, community-based respirologists, spiritual care practitioners	Hospital-to-home care, self-management education; individualised written action plans; ACP discussion; psychosocial and spiritual support; referrals to allied health practitioners	‘Before and after intervention’ observational study over 6 months	Reduced ED visits, hospital admissions and length of hospital stay; improved self-efficacy	Reduced anxiety, improved confidence and willingness to discuss ACP	Significant and sustained cost reduction
Advanced Lung Disease Service^72,85,86^	2013–ongoing	Australia	COPD	Hospital and home-based care, with phone calls	Respiratory and palliative care specialists, psychologists, nurse-led community outreach service	Optimisation of disease-directed management; pharmacological and non-pharmacological breathlessness management; education of self-management; ACP discussion; individualised written action plans and breathlessness management; carer support; psychosocial support; access to ‘Hospital in the Home’; partnership with GP	Observational cohort study over 4 years	Reduced ED visits; significant improvement in breathlessness measured by the numeric rating score at 6 weeks; non-statistical improvement in anxiety and depression	High levels of patient and carer satisfaction	N/A
Munich Breathlessness Service^75,87,88^	2014–2019	Germany	Chronic breathlessness regardless of the underlying causes	Outpatient and community-based care	Respiratory and palliative care specialists, PT, psychologists, SW and nurses	Breathlessness assessment, pharmacological and non-pharmacological management; individualised action plans; self-management; provision of aids; ACP discussion; carer support; psychosocial support	BreathEase RCT 6–8 weeks	Significant improvement in CRQ Mastery and quality of life	Positive impact on self-efficacy and carer support	N/A

ACP, advance care planning; CRQ, Chronic Respiratory Disease Questionnaire; ED, emergency department; GP, general practitioners; MRC, Medical Research Council; N/A, not applicable; OT, occupational therapist; PT, physiotherapist; RCT, randomised controlled trials; SW, social worker.

The Cambridge Breathlessness Intervention Service (CBIS) was founded in 2003 in the department of palliative care at Addenbrooke’s Hospital, a tertiary referral centre in the United Kingdom. It comprises a palliative care specialist (as the service leader) and a clinical specialist physiotherapist or an occupational therapist.^73^ The service focuses on optimisation of severe chronic breathlessness regardless of the underlying causes. They provide clinical assessment predominantly in the patient’s home or via phone calls. The service was developed and remodelled through phase I–III exploratory, feasibility, randomised controlled studies.^77–79^ The phase III fast-track randomised controlled trial was conducted over 4 weeks in people with advanced non-malignant diseases, with COPD being the most common diagnosis.^79,80^ The study demonstrated non-statistical improvements in distress, mastery in chronic breathlessness, and anxiety and depression in patients and carers. The CBIS resulted in minimal higher inpatient costs (£100) after excluding the outliers.^80^ Patients and carers reported a positive impact on their emotions (reduced fear, anxiety and worry) and confidence in breathlessness management. Additionally, consumers highly valued the multi-disciplinary expertise and advice, positive and professional approaches, flexible locations and adequate time for counselling and reassurance.^80^

Based on the CBIS model, the Breathlessness Support Service (BSS) was developed at King’s College Hospital in Southeast London, UK. The BSS served patients with refractory breathlessness (Medical Research Council (MRC) dyspnoea score ⩾2) despite optimisation of the underlying disease, including malignancy, cardiology, respiratory or neurological conditions.^81^ Patients were referred from three large teaching hospitals and general practitioners. Different from the CBIS, the BSS was predominantly an outpatient clinic-based model, combined with home visits and telephone calls between clinics, and led by respiratory and palliative specialists jointly.^81^ The service was extended to involve respiratory specialists to assess and optimise the underlying condition in the initial clinic. Other team members included a clinical nurse specialist and allied health professionals—a physiotherapist, an occupational therapist and a social worker, to facilitate adjustment to daily activities and tailor training and self-management strategies based on the home situation.^81^ The fast-track randomised controlled trial of 105 patients referred to the BSS was conducted between 2010 and 2012.^71^ There was a significant improvement (16%) and difference between the BSS and control groups in mastery of breathlessness measured by Chronic Respiratory Disease Questionnaire (CRQ) at 6 weeks.^71^ The BSS intervention group also reported improvement in secondary outcomes - breathlessness severity, depression and daily activities, although they were not significantly different from the control group. There was no significant reduction in healthcare costs.^71^ Qualitative interviews revealed that patients appreciated the patient-centred holistic approach of the BSS and found the interventions and recommendations acceptable and very helpful.^82^

In 2010, Rocker and Cook implemented the INSPIRED community outreach programme in Canada for patients with advanced COPD.^74^ The service offers home-based care bi-weekly from a respiratory therapist as the care coordinator, a community-based respirologist, and a spiritual care practitioner. The model is underpinned by an approach of continuity of care and non-abandonment.^74^ In addition to provision of personalised breathlessness management, the INSPIRED programme also focuses on proactive in-home ACP as routine practice throughout the disease trajectory.^74^ An observational mixed-methods study of the INSPIRED model suggested improved self-efficacy, confidence and engagement in care over 6 months, as well as significant and sustained cost savings from reduced emergency visits, hospital admissions and length of stay.^83,84^

In Australia, the Advanced Lung Disease Service (ALDS) was established in 2013 at the Royal Melbourne Hospital, a tertiary hospital, for patients with non-malignant respiratory diseases.^72^ The service provides long-term hospital and home-based care and is also founded on an approach of continuity of care and non-abandonment. All patients are introduced to both respiratory and palliative care specialists in the ALDS clinic with respiratory specialists being the primary care provider.^72^ The respiratory specialists in the ALDS clinic have completed basic palliative care training (e.g. clinical diploma or university postgraduate certificate in palliative medicine).^72^ The ALDS also involves a psychologist, a nurse-led community outreach service and telehealth support to allow flexible follow-up tailored to patients’ needs and social situations. The service works collaboratively with ‘Hospital in the Home’, community respiratory and palliative care teams, and aged care assessment services. In particular, the ALDS offers shared care with general practitioners to encourage partnership with primary care.^72^ The ALDS observational cohort study was the first study that showed longer-term results over 4 years. The ALDS results are consistent with those from RCTs and cohort studies, including improved end-of-life care, better access to palliative care specialist services, reduced emergency department visits, and increased engagement in advanced care planning.^72^ Moreover, the mixed-methods prospective cohort study of ALDS patients with COPD showed a clinically significant improvement in breathlessness measured by the numeric rating score at 6 weeks.^85^ Patients and carers reported high levels of satisfaction due to individualised breathlessness education, self-management, continuity of care, and reassurance that an acute episode of breathlessness would pass.^85,86^

Lastly, the Munich Breathlessness Service (MBS) commenced at the Munich University Hospital in 2014.^75^ The MBS provided 6-week, short-term care for patients with refractory breathlessness due to any condition, with COPD being the most common diagnosis (75%). The service was led by palliative care physicians in conjunction with respiratory physicians (who assessed patients in the first appointment and switched to optional based on their needs), physiotherapists, psychologists, social workers and nurses.^75,87^ Patients attended two outpatient MBS appointments in the first and last week and four weekly 60-min community-based physiotherapy sessions in between clinics. Patients’ participation, treatment outcomes, care needs and advanced care planning were reviewed in the second appointment by palliative care clinicians to plan for future management, including referrals to allied health professionals and ongoing palliative care clinics.^75^ Alongside the MBS, the fast-track RCT (BreathEase) of 183 patients was conducted from 2013 to 2015.^88^ The study confirmed significant improvement in CRQ Mastery and CRQ quality of life in the treatment group.^88^ Unfortunately, only half of the participants were able to complete the treatment within 6 weeks and about 12% were lost to follow-up in the treatment group.^75,88^ This reflects the real-world challenge of both delivering care and conducting clinical trials with patients experiencing severe illness and major symptom burden.

These five integrated palliative care services adopt a multi-disciplinary and multi-professional model of care tailored to patients’ complex symptom burden and diverse physical and psychosocial needs.^71–75^ Patients and carers greatly benefited from professional expertise, personalised education, positive reassurance and continuity of care.^80,82,84,85,88^ Studies demonstrated significant improvements in breathlessness, quality of life, confidence in disease and symptom management, as well as symptom-related distress in patients and carers. Beyond the disease and symptom management, the mode of care delivery strengthened patients’ trust, engagement and commitment to ongoing care.^71–75^ These breathlessness services provide a patient-centred and holistic approach that allows time for patients to tell their stories, variation in treatment components, and flexibility around patients’ physical and social needs.^71–75^ Based on the CBIS study, Spathis et al. proposed the Breathing, Thinking, Functioning (BTF) clinical model to explain and address the complex neurophysiological nature of breathlessness and its interaction with patients’ emotions, perception and consequently negative behavioural changes, causing vicious cycles.^89^ Therefore, the multi-modal interventions utilised in these services, integrating respiratory physiotherapy (breathing domain), psychological support (thinking domain), aids and behaviour adjustments (function domain), potentially inform the structure for breathlessness management and education in the future.^75^

### Nurse-led care

Long et al. conducted a prospective, mixed-method pilot study to evaluate the feasibility and effectiveness of an advanced practice nurse-led palliative care programme for patients with advanced stage COPD (GOLD stage III and IV) over 3 months.^90^ The programme aimed to optimise the symptom triad of breathlessness, anxiety and depression. Fifteen patients were enrolled in a tertiary care respiratory clinic. They were provided with individualised pharmacological and non-pharmacological interventions via three outpatient appointments and telephone reviews.^90^ The advanced practice nurse as the care coordinator was able to consult and update the palliative and pulmonary clinicians for the treatment plans.^90^ Patients reported improvement in breathlessness and reduced anxiety and depression even without the use of antidepressants or anxiolytics. They all had positive experiences and wanted to follow-up long term.^90^ Therefore, nurses and allied health professionals have a key role in symptom management and supporting people with COPD.

The HELP-COPD programme was developed in Scotland in 2012–2013 for patients recently admitted for an exacerbation of COPD.^91^ The programme was coordinated by a specialist respiratory nurse (who had experience in respiratory and palliative care) and aimed to offer a holistic care approach via home visits and telephone consultation over 6 months.^91^ Despite the positive feedback, 12 out of 44 (27%) patients withdrew from the study due to illness, death, or loss of contact, indicating the importance of trying to offer supportive care earlier in the illness trajectory.^91^

### Hospice and residential aged care services

Community palliative care services and hospice programmes can expand the reach of palliative care and enable patients to access care locally. These models are diverse in nature but frequently adopt a multi-disciplinary approach to provide comprehensive care. COPD-specific palliative and rehabilitation programmes have been developed in residential aged care and hospices to provide personalised pulmonary rehabilitation and facilitate end-of-life care.^92,93^ The multi-disciplinary geriatrician-led rehabilitation programme and the community hospice programme demonstrated improvement in function capacity, health status and reduction in hospitalisation. For patients in the hospice, they were more likely to receive end-of-life care at home or in the hospice based on their wishes. Notably, symptom management and carers’ stress were the two main reasons for referral to the community hospice programme.^92^ However, the low enrolment rate in the community hospice programme might reflect patients’ and healthcare providers’ hesitance and reluctance in introducing palliative care, and misconceptions about the scope of palliative care management. More recently, a mixed-methods pilot study of the Multidisciplinary Breathlessness Support Service (MBSS) provided evidence for the feasibility and acceptability of hospice-based care.^94^ The MBSS adopted the BTF clinical model to inform its management. Again, it highlighted challenges in referral, implementation and maintenance of services due to lack of clear referral pathways, limited access to primary care practitioners for safe prescribing, as well as patients’ willingness to accept palliative care referrals.

### Home-based palliative care

Home-based palliative care represents a model of delivery which expands upon community and outpatient-based services to deliver palliative care services directly within the person’s home. Many people with COPD struggle to attend traditional consultations due to reduced mobility, lack of readily accessible and affordable transport options, and avoidance of public settings due to exposure to infection risk.^95^ These services aim to reduce the treatment burden for people with COPD and their carers and support people to remain in their homes and communities.

A randomised controlled trial of nurse-led home-based hospice service in the United States, PhoenixCare, which enrolled 192 people with COPD or chronic heart failure who were within the last 2 years of life was effective in reducing symptom distress and unscheduled healthcare utilisation whilst improving physical functioning and quality of life.^96^ Similar programmes piloted in Canada^97^ and Switzerland^98^ were unable to demonstrate effectiveness, yet a larger scale audit in Belgium which included over 58,000 decedents with COPD, of which 644 had received palliative home care demonstrated effectiveness at reducing hospitalisation and increasing odds of dying at home.^99^

### Telemonitoring and telehealth

Whilst services which provide in-home care are promising, they are extremely resource intensive for health services which can limit their capacity. Telehealth and other remote consultations are an alternative and lower-cost means of providing care at home. The use of telehealth for management of COPD is not a novel concept,^100,101^ however the COVID-19 pandemic necessarily increased familiarity and uptake of such services particularly in regards to end-of-life care.

An evaluation of a nurse-led telehealth palliative care intervention in the United States, Project EPIC, which consisted of six sessions mapped against commonly identified palliative care needs was shown to be feasible and acceptable for people with COPD and their carers.^102^ Another feasibility study in Italy delivered follow-up telehealth consultations for people discharged after an exacerbation of COPD with high levels of reported patient satisfaction.^103^ Although these pilot trials are promising, larger studies are necessary to determine effectiveness. Qualitative studies of both patients and clinicians indicate that future models should look towards blended care delivery with telehealth available as a means of providing low-cost and accessible follow-up support, but suggest that telehealth alone is not an adequate substitute for face-to-face consultations.^103,104^

## Conclusion

Palliative care plays a significant role in the care of people with COPD and should be accessible early and concurrently with disease-directed therapies throughout the illness trajectory. This review highlighted the importance of proactive palliative care management and explored the provision of diverse palliative care models through generalist and/or specialist approaches via different delivery methods in a range of settings. The integrated multi-disciplinary services demonstrated their effectiveness, safety and feasibility by achieving improvements in disease and symptom burden, and high levels of satisfaction in patients and caregivers. It should be noted that the models described are often resource intensive and may be challenging to implement in low-resource healthcare services, particularly in low- and middle-income countries where COPD is highly prevalent and burdensome. The challenges of implementing integrated palliative care within low- and middle-income countries are not unique to COPD and cost-effectiveness and scalability remain a challenge even within high-income countries. A successful palliative care model must be practical, accessible, and innovative to respond to individuals’ complex and evolving needs, enhance interdisciplinary and interprofessional partnerships, and to optimise utilisation of local healthcare resources.

## Declarations

**Ethics approval and consent to participate:** Not applicable.

**Consent for publication:** Not applicable.

**Availability of data and materials:** Not applicable.

## References

[1] Agustí A Celli BR Criner GJ , et al. Global initiative for chronic obstructive lung disease 2023 report: GOLD Executive Summary. Eur Respir J 2023; 61: 2300239. 36858443 10.1183/13993003.00239-2023 PMC10066569

[2] Adeloye D Song P Zhu Y , et al. Global, regional, and national prevalence of, and risk factors for, chronic obstructive pulmonary disease (COPD) in 2019: a systematic review and modelling analysis. Lancet Respir Med 2022; 10: 447–458. 35279265 10.1016/S2213-2600(21)00511-7 PMC9050565

[3] Boers E Barrett M Su JG , et al. Global burden of chronic obstructive pulmonary disease through 2050. JAMA Netw Open 2023; 6: e2346598. 10.1001/jamanetworkopen.2023.46598 PMC10704283 38060225

[4] Philip J Gold M Brand C , et al. Facilitating change and adaptation: the experiences of current and bereaved carers of patients with severe chronic obstructive pulmonary disease. J Palliat Med 2014; 17: 421–427. 24502658 10.1089/jpm.2013.0339

[5] Seamark DA Blake SD Seamark CJ , et al. Living with severe chronic obstructive pulmonary disease (COPD): perceptions of patients and their carers. An interpretative phenomenological analysis. Palliat Med 2004; 18: 619–625. 15540670 10.1191/0269216304pm928oa

[6] Ferrell BR Twaddle ML Melnick A , et al. National consensus project clinical practice guidelines for quality palliative care guidelines, 4th edition. J Palliat Med 2018; 21: 1684–1689. 30179523 10.1089/jpm.2018.0431

[7] Young J Donahue M Farquhar M , et al. Using opioids to treat dyspnea in advanced COPD: attitudes and experiences of family physicians and respiratory therapists. Can Fam Physician 2012; 58: e401–407. PMC3395547 22798476

[8] Blinderman CD Homel P Billings JA , et al. Symptom distress and quality of life in patients with advanced chronic obstructive pulmonary disease. J Pain Symptom Manage 2009; 38: 115–123. 19232893 10.1016/j.jpainsymman.2008.07.006

[9] Beernaert K Cohen J Deliens L , et al. Referral to palliative care in COPD and other chronic diseases: a population-based study. Respir Med 2013; 107: 1731–1739. 23810150 10.1016/j.rmed.2013.06.003

[10] Kendzerska T Nickerson JW Hsu AT , et al. End-of-life care in individuals with respiratory diseases: a population study comparing the dying experience between those with chronic obstructive pulmonary disease and lung cancer. Int J Chron Obstruct Pulmon Dis 2019; 14: 1691–1701. 31534323 10.2147/COPD.S210916 PMC6681558

[11] Bloom CI Slaich B Morales DR , et al. Low uptake of palliative care for COPD patients within primary care in the UK. Eur Respir J 2018; 51: 1701879. 29444916 10.1183/13993003.01879-2017 PMC5898942

[12] Disler R Pascoe A Luckett T , et al. Barriers to palliative care referral and advance care planning (ACP) for patients with COPD: a cross-sectional survey of palliative care nurses. Am J Hosp Palliat Care 2022; 39: 169–177. 34013782 10.1177/10499091211018192

[13] Janssen DJA Bajwah S Boon MH , et al. European Respiratory Society clinical practice guideline: palliative care for people with COPD or interstitial lung disease. Eur Respir J 2023; 62: 2202014. 37290789 10.1183/13993003.02014-2022

[bibr14-17534666241310987] Sullivan DR Iyer AS Enguidanos S , et al. Palliative Care Early in the Care Continuum among patients with serious respiratory illness: an official ATS/AAHPM/HPNA/SWHPN Policy Statement. Am J Respir Critic Care Med 2022; 206: e44–e69. 10.1164/rccm.202207-1262ST PMC9799127 36112774

[bibr15-17534666241310987] Holland AE Spathis A Marsaa K , et al. European Respiratory Society Clinical Practice Guideline on symptom management for adults with serious respiratory illness. Eur Respir J 2024: 2400335. 10.1038/npjpcrm.2015.20 38719772

[bibr16-17534666241310987] (GOLD) GIfCOLD. Global strategy for prevention, diagnosis and management of COPD: 2024 report; Global initiative for chronic obstructive lung disease, https://goldcopd.org/wp-content/uploads/2024/02/GOLD-2024_v1.2-11Jan24_WMV.pdf (2024, accessed 1 January 2025).

[17] Yang IA George J McDonald CF , et al. The COPD-X plan: Australian and New Zealand Guidelines for the management of chronic obstructive pulmonary disease 2023. Version 2.73. Lung Foundation Australia’s COPD-X Guidelines Committee, https://copdx.org.au/wp-content/uploads/2024/11/COPD-X-V2.76_FINAL.pdf (2023, accessed 1 January 2025).

[18] Australian Health Ministers’ Advisory Council. National strategic framework for chronic conditions. Canberra: Australian Government, 2017.

[19] National Institute for Health and Care Excellence. Chronic obstructive pulmonary disease in over 16s: diagnosis and management. National Institute for Health and Care Excellence, https://www.nice.org.uk/guidance/ng115/resources/chronic-obstructive-pulmonary-disease-in-over-16s-diagnosis-and-management-pdf-66141600098245 (2018, accessed 1 January 2025). 31211541

[20] Defense DoVADo. VA/DoD clinical practice guideline for the management of chronic obstructive pulmonary disease. U.S. Department of Veterans Affairs, https://www.healthquality.va.gov/guidelines/CD/copd/VADODCOPDCPGFinal508.pdf (2021, accessed 1 January 2025).

[21] Palliative Care Service Development Guidelines, https://palliativecare.org.au/wp-content/uploads/dlm_uploads/2018/02/PalliativeCare-Service-Delivery-2018_web2.pdf (2018, accessed 1 January 2025).

[22] Lanken PN Terry PB Delisser HM , et al. An official American Thoracic Society clinical policy statement: palliative care for patients with respiratory diseases and critical illnesses. Am J Respir Crit Care Med 2008; 177: 912–927. 18390964 10.1164/rccm.200605-587ST

[23] Palliative care services in Australia, https://www.aihw.gov.au/reports/palliative-care-services/palliative-care-services-in-australia/contents/overview (2024, accessed 16 July 2024).

[24] Quill TE Abernethy AP. Generalist plus specialist palliative care—creating a more sustainable model. N Engl J Med 2013; 368: 1173–1175. 23465068 10.1056/NEJMp1215620

[25] Defining your specialist palliative care service, https://palliativecare.org.au/wp-content/uploads/dlm_uploads/2018/05/NSAP-Defining-your-Specialist-PC-Service-new.pdf (2018, accessed 16 July 2024).

[26] Iyer AS Sullivan DR Lindell KO , et al. The role of palliative care in COPD. Chest 2022; 161: 1250–1262. 34740592 10.1016/j.chest.2021.10.032 PMC9131048

[27] Forbat L Johnston N Mitchell I. Defining ‘specialist palliative care’: findings from a Delphi study of clinicians. Aust Health Rev 2020; 44: 313–321. 31248475 10.1071/AH18198

[28] Periyakoil VS Gunten CFV Fischer S , et al. Generalist versus specialist palliative medicine. J Palliat Med 2022; 25: 193–199. 35103529 10.1089/jpm.2021.0644 PMC9022124

[29] Block SD Billings JA. A need for scalable outpatient palliative care interventions. Lancet 2014; 383: 1699–1700. 24559580 10.1016/S0140-6736(13)62676-8

[30] Gardiner C Gott M Ingleton C. Factors supporting good partnership working between generalist and specialist palliative care services: a systematic review. Br J Gen Pract 2012; 62: e353–e362. 10.3399/bjgp12X641474 PMC3338057 22546595

[31] Sullivan DR Iyer AS Reinke LF. Collaborative primary palliative care in serious illness: a pragmatic path forward. Ann Am Thorac Soc 2023; 20: 358–360. 36342447 10.1513/AnnalsATS.202206-556VP PMC9993156

[32] Rocker GM Simpson AC Horton R. Palliative care in advanced lung disease: the challenge of integrating palliation into everyday care. Chest 2015; 148: 801–809. 25742140 10.1378/chest.14-2593

[33] Almagro P Yun S Sangil A , et al. Palliative care and prognosis in COPD: a systematic review with a validation cohort. Int J Chron Obstruct Pulmon Dis 2017; 12: 1721–1729. 28652724 10.2147/COPD.S135657 PMC5473497

[34] Kavanagh E Rowley G Simkiss L , et al. Advance care planning for patients with chronic obstructive pulmonary disease on home non-invasive ventilation: a qualitative study exploring barriers, facilitators and patients’ and healthcare professionals’ recommendations. Palliat Med 2023; 37: 1413–1423. 37698008 10.1177/02692163231192130

[35] Han MK Martinez CH Au DH , et al. Meeting the challenge of COPD care delivery in the USA: a multiprovider perspective. Lancet Respir Med 2016; 4: 473–526. 27185520 10.1016/S2213-2600(16)00094-1

[36] Meehan E Foley T Kelly C , et al. Advance care planning for individuals with chronic obstructive pulmonary disease: a scoping review of the literature. J Pain Symptom Manage 2020; 59: 1344–1361. 31837455 10.1016/j.jpainsymman.2019.12.010

[37] Tavares N Jarrett N Hunt K , et al. Palliative and end-of-life care conversations in COPD: a systematic literature review. ERJ Open Res 2017; 3: 20170427. 10.1183/23120541.00068-2016 PMC5407435 28462236

[38] Sudore RL Lum HD You JJ , et al. Defining advance care planning for adults: a consensus definition from a Multidisciplinary Delphi Panel. J Pain Symptom Manage 2017; 53: 821–832.e821. 10.1016/j.jpainsymman.2016.12.331 PMC5728651 28062339

[39] Janssen DJA Engelberg RA Wouters EFM , et al. Advance care planning for patients with COPD: past, present and future. Patient Educ Counsel 2012; 86: 19–24. 10.1016/j.pec.2011.01.007 21316899

[40] Ora L Mannix J Morgan L , et al. Chronic obstructive pulmonary disease and advance care planning: A synthesis of qualitative literature on patients’ experiences. Chronic Illn 2022; 18: 221–233. 33573389 10.1177/1742395321990109

[41] Teno JM Gruneir A Schwartz Z , et al. Association between advance directives and quality of end-of-life care: a national study. J Am Geriatr Soc 2007; 55: 189–194. 17302654 10.1111/j.1532-5415.2007.01045.x

[42] Rose EK O’Connor J. Addressing advance care planning in patients with COPD. Chest 2022; 161: 676–683. 34762924 10.1016/j.chest.2021.10.037

[43] Patel K Janssen DJ Curtis JR. Advance care planning in COPD. Respirology 2012; 17: 72–78. 22008225 10.1111/j.1440-1843.2011.02087.x

[44] Detering KM Hancock AD Reade MC , et al. The impact of advance care planning on end of life care in elderly patients: randomised controlled trial. BMJ 2010; 340: c1345. 10.1136/bmj.c1345 PMC2844949 20332506

[45] Gardiner C Gott M Small N , et al. Living with advanced chronic obstructive pulmonary disease: patients concerns regarding death and dying. Palliat Med 2009; 23: 691–697. 19825897 10.1177/0269216309107003

[46] Curtis JR. Palliative and end-of-life care for patients with severe COPD. Eur Respir J 2008; 32: 796–803. 17989116 10.1183/09031936.00126107

[47] McNeely PD Hébert PC Dales RE , et al. Deciding about mechanical ventilation in end-stage chronic obstructive pulmonary disease: how respirologists perceive their role. CMAJ 1997; 156: 177–183. 9012718 PMC1226905

[48] Disler R Cui Y Luckett T , et al. Respiratory nurses have positive attitudes but lack confidence in advance care planning for chronic obstructive pulmonary disease: online survey. J Hosp Palliat Nurs 2021; 23: 442–454. 34369423 10.1097/NJH.0000000000000778

[49] Seamark D Blake S Seamark C , et al. Is hospitalisation for COPD an opportunity for advance care planning? A qualitative study. Prim Care Respir J 2012; 21: 261–266. 22596245 10.4104/pcrj.2012.00032 PMC6547950

[50] Rajnoveanu RM Rajnoveanu AG Fildan AP , et al. Palliative care initiation in chronic obstructive pulmonary disease: prognosis-based, symptoms-based or needs-based? Int J Chron Obstruct Pulmon Dis 2020; 15: 1591–1600. 32694913 10.2147/COPD.S254104 PMC7340370

[51] Uronis HE Currow DC Abernethy AP. Palliative management of refractory dyspnea in COPD. Int J COPD 2006; 1: 289–304. 10.2147/copd.2006.1.3.289 PMC2707160 18046866

[52] Gysels M Higginson IJ. The experience of breathlessness: the social course of chronic obstructive pulmonary disease. J Pain Symptom Manage 2010; 39: 555–563. 20303029 10.1016/j.jpainsymman.2009.08.009

[53] Frostad A Soyseth V Haldorsen T , et al. Respiratory symptoms and long-term cardiovascular mortality. Respir Med 2007; 101: 2289–2296. 17681463 10.1016/j.rmed.2007.06.023

[54] Dyspnea. Mechanisms, assessment, and management: a consensus statement. American Thoracic Society. Am J Respir Crit Care Med 1999; 159: 321–340. 9872857 10.1164/ajrccm.159.1.ats898

[55] Gysels M Higginson IJ. Access to services for patients with chronic obstructive pulmonary disease: the invisibility of breathlessness. J Pain Symptom Manag 2008; 36: 451–460. 10.1016/j.jpainsymman.2007.11.008 18495412

[56] Johnson MJ Yorke J Hansen-Flaschen J , et al. Towards an expert consensus to delineate a clinical syndrome of chronic breathlessness. Eur Respir J 2017; 49: 20170525. 10.1183/13993003.02277-2016 28546269

[57] Rocker G Horton R Currow D , et al. Palliation of dyspnoea in advanced COPD: revisiting a role for opioids. Thorax 2009; 64: 910–915. 19786716 10.1136/thx.2009.116699

[58] Maddocks M Lovell N Booth S , et al. Palliative care and management of troublesome symptoms for people with chronic obstructive pulmonary disease. Lancet 2017; 390: 988–1002. 28872031 10.1016/S0140-6736(17)32127-X

[59] McNamara RJ Epsley C Coren E , et al. Singing for adults with chronic obstructive pulmonary disease (COPD). Cochrane Database Syst Rev 2017; 12: Cd012296. 10.1002/14651858.CD012296.pub2 PMC5835013 29253921

[60] Ly L Philip J Hudson P , et al. Singing for people with advance chronic respiratory diseases: a qualitative meta-synthesis. Biomedicines 2022; 10: 20220826. 10.3390/biomedicines10092086 PMC9495573 36140187

[61] Verberkt CA van den Beuken-van Everdingen MHJ Schols JMGA , et al. Effect of sustained-release morphine for refractory breathlessness in chronic obstructive pulmonary disease on health status: a randomized clinical trial. JAMA Intern Med 2020; 180: 1306–1314. 32804188 10.1001/jamainternmed.2020.3134 PMC7432282

[62] Johnson MJ Currow DC. Opioids for breathlessness: a narrative review. BMJ Support Palliat Care 2020; 10: 287–295. 10.1136/bmjspcare-2020-002314 32620683

[63] Chen X Treanor D Le B , et al. Gaps in opioid prescription for severe breathlessness in hospitalized patients with chronic obstructive pulmonary disease or interstitial lung disease. J Pain Symptom Manage 2020; 60: e36–e39. 10.1016/j.jpainsymman.2020.05.017 32446973

[64] Goërtz YMJ Spruit MA Van’t Hul AJ , et al. Fatigue is highly prevalent in patients with COPD and correlates poorly with the degree of airflow limitation. Ther Adv Respir Dis 2019; 13: 1753466619878128. 10.1177/1753466619878128 PMC6767724 31558115

[65] Avdeev SN Vizel AA Abrosimov VN , et al. Management of cough in patients with chronic obstructive pulmonary disease: results of the Multicenter Randomized Placebo-Controlled Clinical Trial. Int J Chron Obstruct Pulmon Dis 2021; 16: 1243–1253. 33981141 10.2147/COPD.S292109 PMC8107011

[66] Ebadi Z Goërtz YMJ Van Herck M , et al. The prevalence and related factors of fatigue in patients with COPD: a systematic review. Eur Respir Rev 2021; 30: 20210413. 10.1183/16000617.0298-2020 PMC9489028 33853886

[67] Nakken N Janssen DJA van den Bogaart EHA , et al. Informal caregivers of patients with COPD: home sweet home? Eur Respir Rev 2015; 24: 498. 26324811 10.1183/16000617.00010114 PMC9487697

[68] Trivedi RB Bryson CL Udris E , et al. The influence of informal caregivers on adherence in COPD patients. Ann Behav Med 2012; 44: 66–72. 22422104 10.1007/s12160-012-9355-8

[69] Giacomini M DeJean D Simeonov D , et al. Experiences of living and dying with COPD: a systematic review and synthesis of the qualitative empirical literature. Ont Health Technol Assess Ser 2012; 12: 1–47. PMC3384365 23074423

[70] Hasson F Spence A Waldron M , et al. Experiences and needs of bereaved carers during palliative and end-of-life care for people with chronic obstructive pulmonary disease. J Palliat Care 2009; 25: 157–163. 19824276

[71] Higginson IJ Bausewein C Reilly CC , et al. An integrated palliative and respiratory care service for patients with advanced disease and refractory breathlessness: a randomised controlled trial. Lancet Respir Med 2014; 2: 979. 25465642 10.1016/S2213-2600(14)70226-7

[bibr72-17534666241310987] Smallwood N Thompson M Warrender-Sparkes M , et al. Integrated respiratory and palliative care may improve outcomes in advanced lung disease. ERJ Open Res 2017; 4(1): 00102-2017. 10.1183/23120541.00102-2017. PMC5912931 29707561

[bibr73-17534666241310987] Booth S Moffat C Farquhar M , et al. Developing a breathlessness intervention service for patients with palliative and supportive care needs, irrespective of diagnosis. J Palliat Care 2011; 27: 28–36. 21510129

[bibr74-17534666241310987] Rocker GM Cook D. ‘INSPIRED’ approaches to better care for patients with advanced COPD. Clin Invest Med 2013; 36: E114–E120. 10.25011/cim.v36i3.19721 23739664

[75] Bausewein C Schunk M Schumacher P , et al. Breathlessness services as a new model of support for patients with respiratory disease. Chron Respir Dis 2018; 15: 48–59. 28718321 10.1177/1479972317721557 PMC5802660

[76] Philip J Crawford G Brand C , et al. A conceptual model: redesigning how we provide palliative care for patients with chronic obstructive pulmonary disease. Palliat Support Care 2018; 16: 452–460. 28560949 10.1017/S147895151700044X

[77] Farquhar M Higginson IJ Fagan P , et al. Results of a pilot investigation into a complex intervention for breathlessness in advanced chronic obstructive pulmonary disease (COPD): brief report. Palliat Support Care 2010; 8: 143–149. 20307365 10.1017/S1478951509990897

[78] Booth S Farquhar M Gysels M , et al. The impact of a breathlessness intervention service (BIS) on the lives of patients with intractable dyspnea: a qualitative phase 1 study. Palliat Support Care 2006; 4: 287–293. 17066970 10.1017/s1478951506060366

[79] Farquhar MC Prevost AT McCrone P , et al. Study protocol: phase III single-blinded fast-track pragmatic randomised controlled trial of a complex intervention for breathlessness in advanced disease. Trials 2011; 12: 130. 21599896 10.1186/1745-6215-12-130 PMC3114770

[80] Farquhar MC Prevost AT McCrone P , et al. The clinical and cost effectiveness of a Breathlessness Intervention Service for patients with advanced non-malignant disease and their informal carers: mixed findings of a mixed method randomised controlled trial. Trials 2016; 17: 185. 27044249 10.1186/s13063-016-1304-6 PMC4820876

[81] Bausewein C Jolley C Reilly C , et al. Development, effectiveness and cost-effectiveness of a new out-patient Breathlessness Support Service: study protocol of a phase III fast-track randomised controlled trial. BMC Pulm Med 2012; 12: 58. 22992240 10.1186/1471-2466-12-58 PMC3517322

[82] Gysels M Reilly CC Jolley CJ , et al. How does a new breathlessness support service affect patients? Eur Respir J 2015; 46: 1515–1518. 26381516 10.1183/13993003.00751-2015

[83] Young J Simpson AC Demmons J , et al. Evaluating the impacts of INSPIRED : a new outreach program for patients and families living with advanced chronic obstructive pulmonary disease (COPD). American Journal of Respiratory and Critical Care Medicine, 2012. 10.1164/ajrccm-conference.2012.185.1_MeetingAbstracts.A3732

[84] Rocker GM Verma JY. ‘INSPIRED’ COPD Outreach Program™: doing the right things right. Clin Invest Med 2014; 37: E311–E319. 25282137

[85] Qian MYY Politis J Thompson M , et al. Individualized breathlessness interventions may improve outcomes in patients with advanced COPD. Respirology 2018; 23: 1146–1151. 29763515 10.1111/resp.13324

[86] Smallwood N Moran T Thompson M , et al. Integrated respiratory and palliative care leads to high levels of satisfaction: a survey of patients and carers. BMC Palliat Care 2019; 18: 7. 30660204 10.1186/s12904-019-0390-0 PMC6339689

[87] Schunk M Berger U Le L , et al. BreathEase: rationale, design and recruitment of a randomised trial and embedded mixed-methods study of a multiprofessional breathlessness service in early palliative care. ERJ Open Res 2021; 7: 20211018. 10.1183/23120541.00228-2020 PMC8521025 34671668

[88] Schunk M Le L Syunyaeva Z , et al. Effectiveness of a specialised breathlessness service for patients with advanced disease in Germany: a pragmatic fast-track randomised controlled trial (BreathEase). Eur Respir J 2021; 58: 20210826. 10.1183/13993003.02139-2020 33509957

[89] Spathis A Booth S Moffat C , et al. The Breathing, Thinking, Functioning clinical model: a proposal to facilitate evidence-based breathlessness management in chronic respiratory disease. NPJ Prim Care Respir Med 2017; 27: 27. 28432286 10.1038/s41533-017-0024-z PMC5435098

[90] Long MB Bekelman DB Make B. Improving quality of life in chronic obstructive pulmonary disease by integrating palliative approaches to dyspnea, anxiety, and depression. J Hosp Palliat Nurs 2014; 16. 10.1038/npjpcrm.2015.20

[91] Buckingham S Kendall M Ferguson S , et al. HELPing older people with very severe chronic obstructive pulmonary disease (HELP-COPD): mixed-method feasibility pilot randomised controlled trial of a novel intervention. NPJ Prim Care Respir Med 2015; 25: 15020. 26028347 10.1038/npjpcrm.2015.20 PMC4532154

[92] Iupati SP Ensor BR. Do community hospice programmes reduce hospitalisation rate in patients with advanced chronic obstructive pulmonary disease? Intern Med J 2016; 46: 295–300. 26549020 10.1111/imj.12947

[93] van Dam van Isselt EF Spruit M Groenewegen-Sipkema KH , et al. Geriatric rehabilitation for patients with advanced chronic obstructive pulmonary disease: a naturalistic prospective cohort study on feasibility and course of health status. Chron Respir Dis 2014; 11: 111–119. 24728657 10.1177/1479972314529674

[94] Drury A Goss J Afolabi J , et al. A mixed methods evaluation of a Pilot Multidisciplinary Breathlessness Support Service. Eval Rev 2023; 47: 820–870. 37014066 10.1177/0193841X231162402 PMC10492442

[95] Sav A Kendall E McMillan SS , et al. ‘You say treatment, I say hard work’: treatment burden among people with chronic illness and their carers in Australia. Health Soc Care Community 2013; 21: 665–674. 23701664 10.1111/hsc.12052

[96] Aiken LS Butner J Lockhart CA , et al. Outcome evaluation of a randomized trial of the PhoenixCare intervention: program of case management and coordinated care for the seriously chronically ill. J Palliat Med 2006; 9: 111–126. 16430351 10.1089/jpm.2006.9.111

[97] Horton R Rocker G Dale A , et al. Implementing a palliative care trial in advanced COPD: a feasibility assessment (the COPD IMPACT study). J Palliat Med 2013; 16: 67–73. 23317322 10.1089/jpm.2012.0285 PMC3546432

[98] Weber C Stirnemann J Herrmann F , et al. 111 Can early introduction of specialized palliative care limit intensive care, emergency and hospital admissions in patients with severe and very severe COPD? A pilot randomized study. CHEST 2017; 151: A6. 10.1186/1472-684X-13-47 PMC4448287 25927907

[99] Scheerens C Faes K Pype P , et al. Earlier palliative home care is associated with patient-centred medical resource utilisation and lower costs in the last 30 days before death in COPD: a population-level decedent cohort study. Eur Respir J 2020; 55: 1901139. 32108048 10.1183/13993003.01139-2019

[100] Segrelles Calvo G Gómez-Suárez C Soriano JB , et al. A home telehealth program for patients with severe COPD: the PROMETE study. Respir Med 2014; 108: 453–462. 24433744 10.1016/j.rmed.2013.12.003

[101] Soriano JB García-Río F Vázquez-Espinosa E , et al. A multicentre, randomized controlled trial of telehealth for the management of COPD. Respir Med 2018; 144: 74–81. 30366588 10.1016/j.rmed.2018.10.008

[102] Iyer AS Wells RD Dionne-Odom JN , et al. Project EPIC (Early Palliative Care in COPD): a formative and summative evaluation of the EPIC Telehealth Intervention. J Pain Symptom Manage 2023; 65: 335–347.e333. 10.1016/j.jpainsymman.2022.11.024 PMC10023469 36496113

[103] Vitacca M Comini L Tabaglio E , et al. Tele-assisted palliative homecare for advanced chronic obstructive pulmonary disease: a feasibility study. J Palliat Med 2019; 22: 173–178. 10.1038/npjpcrm.2015.20 30256709

[104] Pasanen L Le Gautier R Wong A , et al. Telehealth in outpatient delivery of palliative care: a qualitative study of patient and physician views. Palliat Support Care. Epub ahead of print 2022: 1–8. DOI: 10.1017/s1478951522000670. 35818898

